# Enhanced activity of ornithine decarboxylase of the ileum in rats by bracken fern (Pteridium aquilinum).

**DOI:** 10.1038/bjc.1983.187

**Published:** 1983-08

**Authors:** S. Hosaka, H. Nagayama, I. Hirono, M. Haga


*Br. J. Cancer* (1983), **48**, 311–314

### Short Communication

### Enhanced activity of ornithine decarboxylase of the ileum in rats by bracken fern (*Pteridium aquilinum*)

S. Hosaka, H. Nagayama[1], I. Hirono & M. Haga[2]

*Department of Carcinogenesis and Cancer Susceptibility, Institute of Medical Science, University of Tokyo, 4-6-1, Shirokanedai, Minato-ku, Tokyo, [1]Department of Pediatrics, Research Institute for Tuberculosis and Cancer, Tohoku University, 4-1, Seiryo-machi, Sendai and [2]Department of Pharmaceutical Science, Higashi Nippon Gakuen University, Ishikari, Hokkaido, Japan.*

It has been known that ileal and urinary bladder tumours were induced by feeding bracken fern to rats and cows (Evans & Manson, 1965; Pamukcu *et al.*, 1967; Price & Pamukcu, 1968; Hirono *et al.*, 1970). Nevertheless, the isolation of a carcinogenic substance present in bracken fern has not yet been successful. Pamukcu *et al.* (1980) reported that quercetin which is contained in bracken fern was carcinogenic for intestine and urinary bladder in non-inbred rats derived from the Norwegian strain. However, Hirono *et al.* (1981) could not confirm the carcinogenicity of quercetin in ACI rats, which were susceptible to the carcinogenicity of bracken fern (Hirono *et al.*, 1970). Accordingly, they concluded that the carcinogenicity of bracken fern could not be attributable to quercetin.

Since the absence of acute toxicity of bracken fern in laboratory rodents has been regarded as one reason for the difficulty in isolating bracken fern carcinogen(s), attention has been given to biochemical changes that might be induced by the carcinogen in target organs. Due to the increased activity of ornithine decarboxylase (ODC) in target organs following the administration of tumour promoters (O'Brien *et al.*, 1975; O'Brien, 1976; Fujiki *et al.*, 1979) and carcinogens (Ball *et al.*, 1976; O'Brien, 1976; Scalabrino *et al.*, 1978; Olson & Russell, 1979; Matsushima & Bryan, 1980; Takano *et al.*, 1981), we examined ODC activity of the ileum of rats fed a diet containing bracken fern in an attempt to use it as an indicator for the isolation of carcinogenic or tumour-promoting substances in bracken fern.

Inbred strain ACI rats were obtained from our institute and breeding was carried out in our laboratory. Both sexes of rats at 5 weeks of age were used. They were freely given a diet containing bracken fern and water throughout the experimental period and were killed by ether between 10 and 11 a.m. to avoid circadian rhythm variations (Fujimoto *et al.*, 1978). The fresh mature bracken fern, collected in August, in 1979 in Hokkaido, Japan, was dried and powdered. Then, the material was mixed with rat basal diet CE-2 (CLEA, Japan Inc., Tokyo) at a concentration of 30%.

Ileum, jejunum and descending colon were opened lengthwise and rinsed with cold 0.9% NaCl solution to remove debris. The mucosas were scraped off with a spatula and the tissues were homogenized in 2 ml of 50 mM sodium phosphate buffer (pH 7.2) containing 0.1 mM pyridoxal phosphate and 0.1 mM EDTA. The supernatant fraction obtained after centrifugation at 28000 *g* for 50 min at 2° was used for the determination of enzyme activity.

Ornithine decarboxylase activity was determined by measuring the release of $^{14}CO_2$ from DL-[1-$^{14}$C]ornithine hydrochloride (56 mCi mmol$^{-1}$, Amersham Int. Ltd.) as described by Russell and Snyder (1968). The assay mixture contained 50 mM sodium phosphate (pH 7.2), 0.2 mM pyridoxal phosphate, 5.2 mM dithiothreitol, 1 mM EDTA, 0.5 mM L-ornithine containing 0.5 $\mu$Ci of DL-[1-$^{14}$C]ornithine hydrochloride, and 200 $\mu$l of mucosal extract ($\sim$1 mg protein) in a final volume of 0.6 ml. Incubation was carried out for 30 min at 37° and then the reaction was stopped by adding 0.5 ml of 2 M citric acid. Protein content of mucosal extract was determined by the Lowry method.

Figure 1 shows the changes in ODC activity of the ileum in female rats fed bracken diet. The activity was maximal 1 week after the feeding of bracken diet and a significant difference in the ODC activity was seen between the group fed bracken diet and the control ($P<0.001$, by $t$ test). Figure 2 shows the enhanced ODC activity of the ileum in male rats fed bracken diet. The activity was significantly enhanced by the feeding of bracken diet for 1 week ($P<0.01$, by $t$ test) and the high activity level of ODC remained for at least 2

Correspondence S. Hosaka

Received 24 February 1983; accepted 3 May 1983.

© The Macmillan Press Ltd., 1983

**Figure 1** Changes in ileal ODC activity in female rats fed bracken diet. (○) 30% bracken diet; (●) basal diet. Each point represents the mean ±s.d. of assays made with 3–6.

**Figure 2** Changes in ileal ODC activity in male rats fed bracken diet. (○) 30% bracken diet; (●) basal diet. Each point represents the mean ±s.d. of assays made with 3–5 rats.

weeks. A dose-response curve of ODC activity in female rats fed diets containing 5, 15 and 30% of bracken is shown in Figure 3. The activity increased with the increase in the concentration of bracken fern in the diet. An increase of activity of about 2 to 3-fold in the jejunum was observed in rats of both sexes 1, 2 and 7 weeks after the administration of the bracken diet. However, there were no significant differences in the jejunal ODC activity between the group fed bracken diet and the control. ODC activities of colon and liver in male rats fed bracken diet for 1 week were the same as

**Figure 3** Dose-response curve of ileal ODC activity in female rats fed diets containing 5, 15 and 30% of bracken. Each point represents the mean ±s.d. of assays made with 3 rats. Rats were killed after 1 week.

those in rats fed basal diet. In addition, administration of a diet containing boiling water extract of bracken fern, which was shown to be carcinogenic for rats (Hirono *et al.*, 1978), increased ODC activity of the ileum in animals (unpublished data).

There are several reports of the enhancement of ODC activities in the target organ by a single administration of carcinogen to animals (O'Brien, 1976; Olson & Russell, 1979; Matsushima & Bryan, 1980; Takano *et al.*, 1981) or feeding of a diet containing carcinogen (Ball *et al.*, 1976; Scalabrino *et al.*, 1979). Ball *et al.* (1976) reported that administration of an intestinal carcinogen, dimethylhydrazine, led to a large increase in colonic ODC activity but did not affect the ODC in liver: on the contrary, a liver carcinogen, acetylaminofluorene, caused a manifold increase in liver ODC but not that of the colon. ODC activity of urinary bladder or colon was also enhanced by the topical administration of vesical or colonic carcinogen, respectively (Matsushima & Bryan, 1980; Takano *et al.*, 1981). O'Brien (1976) showed that carcinogenic hydrocarbons caused the induction of epidermal ODC two or 3 days after application of the carcinogens and considered that environmental carcinogens as well as tumour promoters could be detected in the ODC induction system.

Taken together with these reports, we considered that the elevated ODC activity of the ileum which was caused by the administration of bracken diet to ACI rats may represent a biochemical characteristic occurring at an early stage in the process of bracken carcinogenesis and will be useful as an index to the isolation of carcinogen or tumour promoter contained in bracken fern.

### References

BALL, W.J., Jr., SALSER, J.S. & BALIS, M.E. (1976). Biochemical changes in preneoplastic rodent intestines. *Cancer Res.*, **36**, 2686.

EVANS, I.A. & MASON, J. (1965). Carcinogenic activity of bracken. *Nature*, **208**, 913.

FUJIKI, H., MORI, M., NAKAYASU, M., TERADA, M. & SUGIMURA, T. (1979). A possible naturally occurring tumour promoter, Teleocidin B from *streptomyces*. *Biochem. Biophys. Res. Commun.*, **90**, 976.

FUJIMOTO, M., KANAYA, A., NAKABOU, Y. & HAGIHIRA, H. (1978). Circadian rhythm in the ornithine decarboxylase activity of rat small intestine. *J. Biochem.* (Tokyo), **83**, 237.

HIRONO, I., SHIBUYA, C., FUSHIMI, K. & HAGA, M. (1970). Studies on carcinogenic properties of bracken, *Pteridium aquilinum*. *J. Natl Cancer Inst.*, **45**, 179.

HIRONO, I., USHIMARU, Y., KATO, K., MORI, H. & SASAOKA, I. (1978). Carcinogenicity of boiling water extract of bracken, *Pteridium aquilinum*. *Gann*, **69**, 383.

HIRONO, I., UENO, I., HOSAKA, S., & 4 others (1981). Carcinogenicity examination of quercetin and rutin in ACI rats. *Cancer Lett.*, **13**, 15.

MATSUSHIMA, M. & BRYAN, G.T. (1980). Early induction of mouse urinary bladder ornithine decarboxylase activity of rodent vesical carcinogens. *Cancer Res.*, **40**, 1897.

O'BRIEN, T.G., SIMSIMAN, R.C. & BOUTWELL, R.K. (1975). Induction of the polyamine-biosynthetic enzymes in mouse epidermis by tumour-promoting agents. *Cancer Res.*, **35**, 1662.

O'BRIEN, T.G. (1976). The induction of ornithine decarboxylase as an early, possibly obligatory, event in mouse skin carcinogenesis. *Cancer Res.*, **36**, 2644.

OLSON, J.W. & RUSSELL, D.H. (1979). Prolonged induction of hepatic ornithine decarboxylase and its relation to cyclic adenosine 3′:5′-monophosphate dependent protein kinase activation after a single administration of diethylinitrosamine. *Cancer Res.*, **39**, 3074.

PAMUKCU, A.M., GOKSOY, S.K. & PRICE, J.M. (1967). Urinary bladder neoplasms induced by feeding bracken fern (*Pteris aquilina*) to cows. *Cancer Res.*, **27**, 917.

PAMUKCU, A.M., YALCINER, S., HATCHER, J.F. & BRYAN, G.T. (1980). Quercetin, a rat intestinal and urinary bladder carcinogen present in bracken fern (*Pteridium aquilinum*). *Cancer Res.*, **40,** 3468.

PRICE, J.M. & PAMUKCU, A.M. (1968). The induction of neoplasms of the urinary bladder of the cow and the small intestine of the rat by feeding bracken fern (*Pteris aquilina*). *Cancer Res.*, **28,** 2247.

RUSSELL, D. & SNYDER, S. (1968). Amine synthesis in rapidly growing tissues: ornithine decarboxylase activity in regenerating rat liver, chick embryo, and various tumours. *Proc. Natl Acad. Sci. USA*, **60,** 1420.

SCALABRINO, G., PÖSÖ, H., HÖLTTÄ, E., HANNONEN, P., KALLIO, A. & JÄNNE, J. (1978). Synthesis and accumulation of polyamines in rat liver during chemical carcinogenesis. *Int. J. Cancer*, **21,** 239.

TAKANO, S., MATSUSHIMA, M., ERTÜRK, E. & BRYAN, G.T. (1981). Early induction of rat colonic epithelial ornithine and S-adenosyl-L-methionine decarboxylase activities by N-methyl-N′-nitro-N-nitrosoguanidine or bile salts. *Cancer Res.*, **41,** 624.


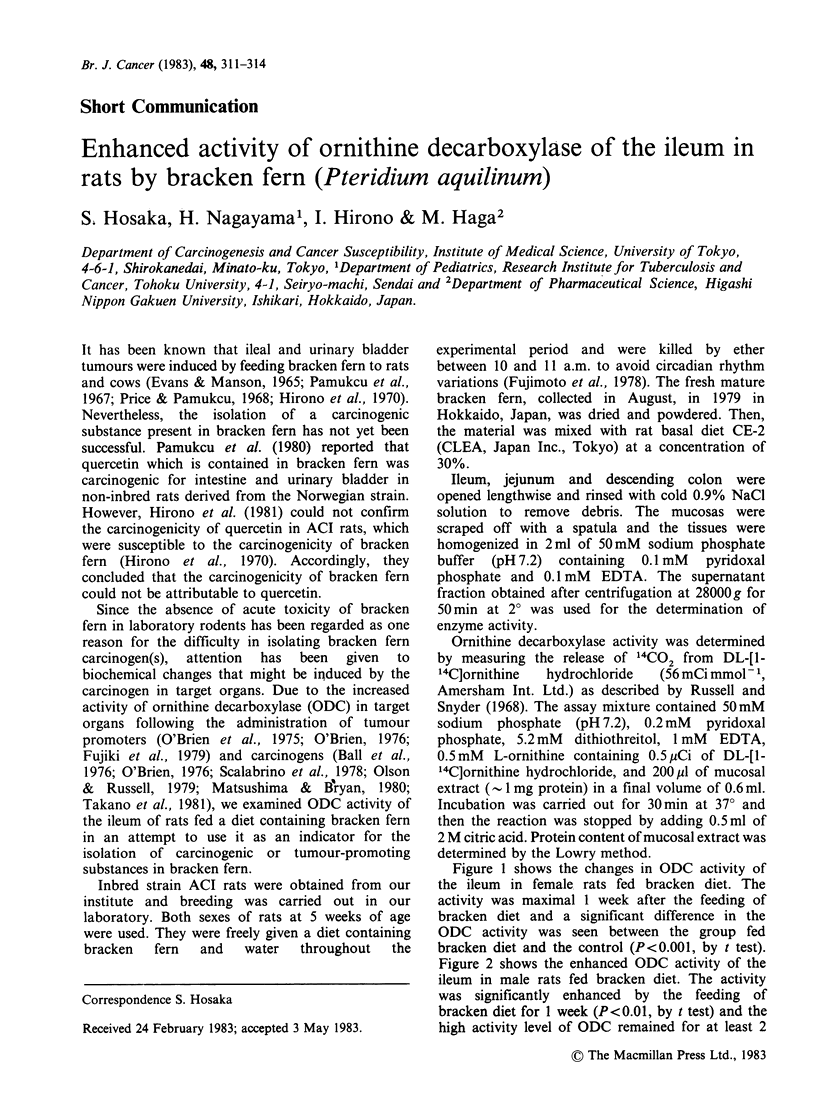

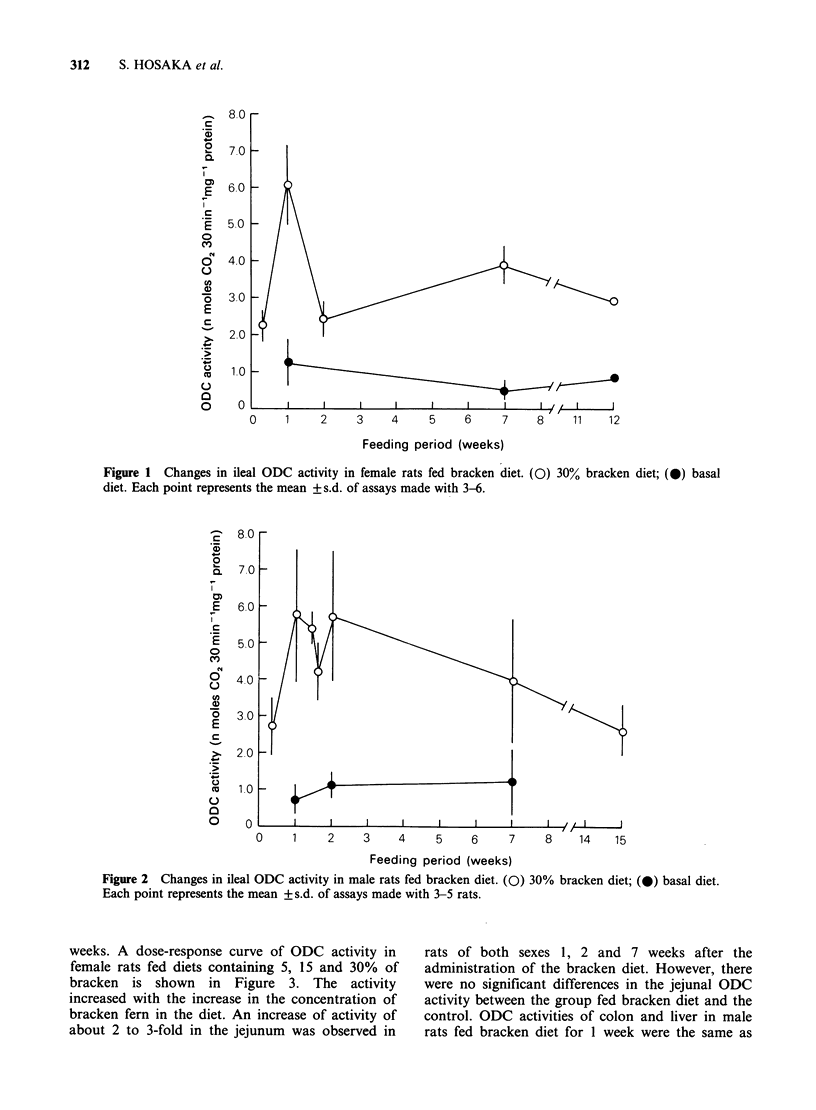

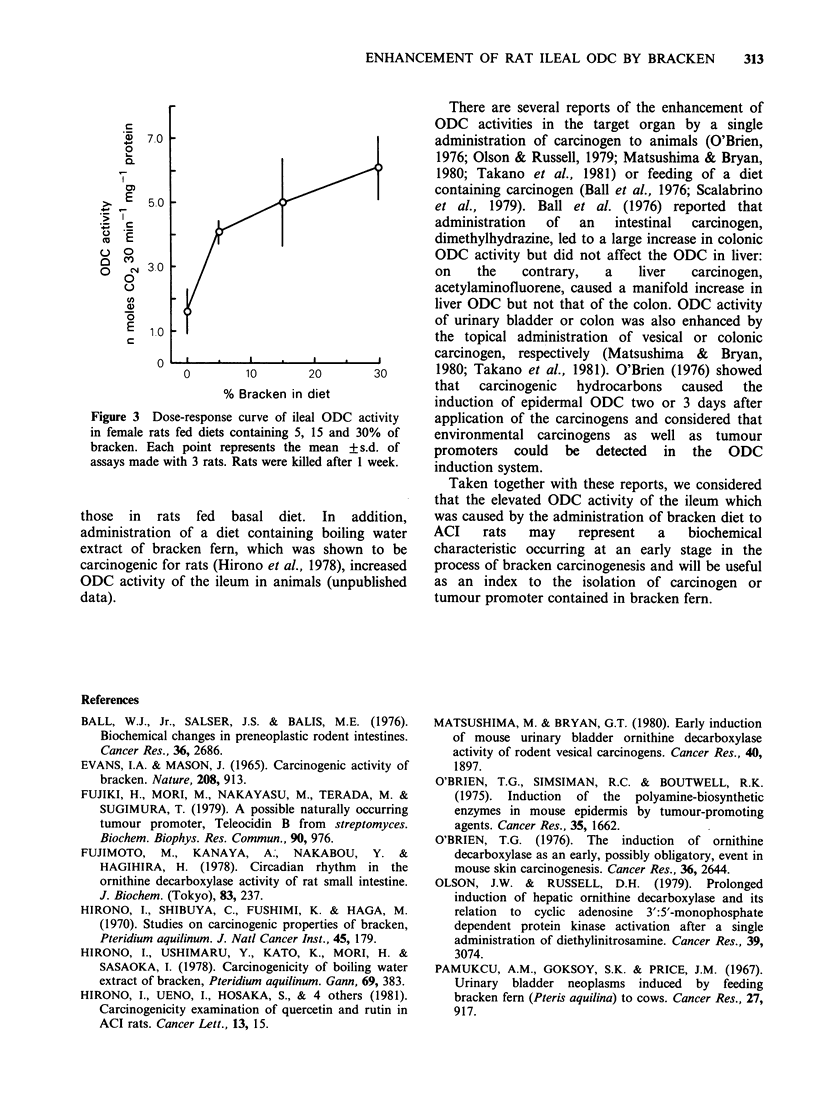

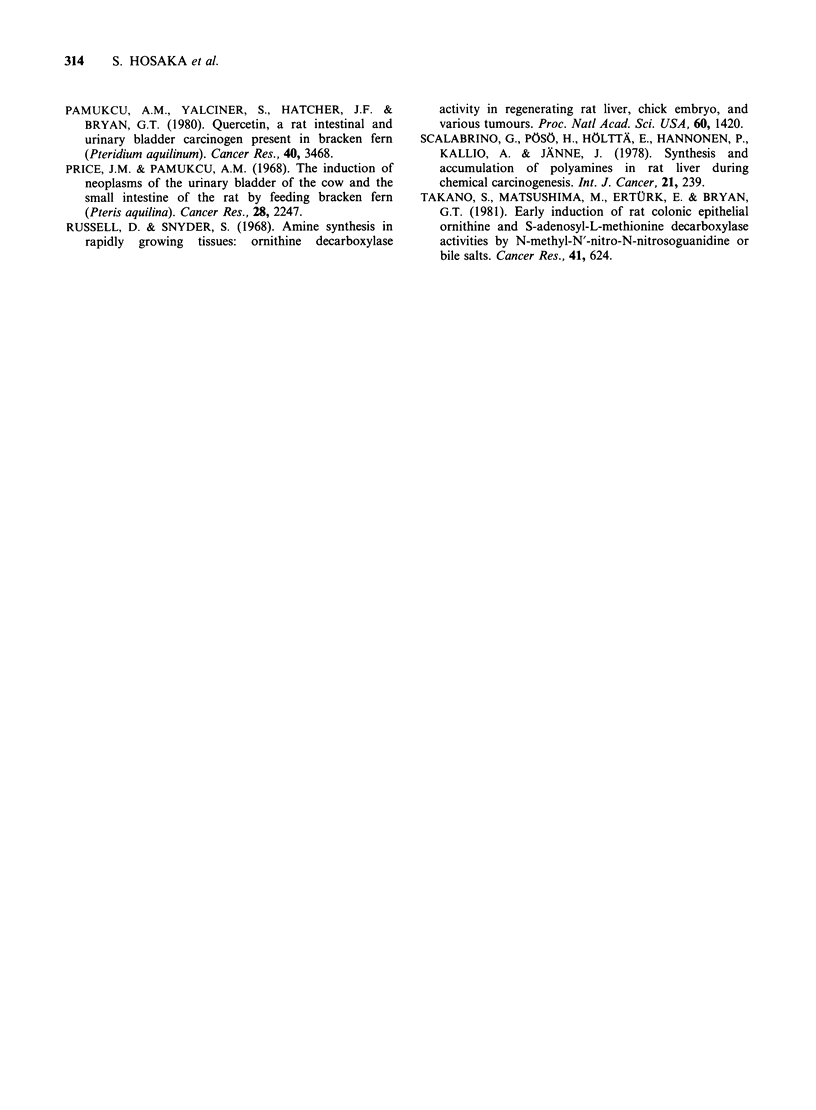


## References

[OCR_00320] Ball W. J., Salser J. S., Balis M. E. (1976). Biochemical changes in preneoplastic rodent intestines.. Cancer Res.

[OCR_00325] Evans I. A., Mason J. (1965). Carcinogenic activity of bracken.. Nature.

[OCR_00329] Fujiki H., Mori M., Nakayasu M., Terada M., Sugimura T. (1979). A possible naturally occurring tumor promoter, teleocidin B from Streptomyces.. Biochem Biophys Res Commun.

[OCR_00337] Fujimoto M., Kanaya A., Nakabou Y., Hagihira H. (1978). Circadian rhythm in the ornithine decarboxylase activity of rat small intestine.. J Biochem.

[OCR_00341] Hirono I., Shibuya C., Fushimi K., Haga M. (1970). Studies on carcinogenic properties of bracken, Pteridium aquilinum.. J Natl Cancer Inst.

[OCR_00346] Hirono I., Ushimaru Y., Kato K., Mori H., Sasaoka I. (1978). Carcinogenicity of boiling water extract of bracken, Pteridium aquilinum.. Gan.

[OCR_00355] Matsushima M., Bryan G. T. (1980). Early induction of mouse urinary bladder ornithine decarboxylase activity by rodent vesical carcinogens.. Cancer Res.

[OCR_00361] O'Brien T. G., Simsiman R. C., Boutwell R. K. (1975). Induction of the polyamine-biosynthetic enzymes in mouse epidermis by tumor-promoting agents.. Cancer Res.

[OCR_00367] O'Brien T. G. (1976). The induction of ornithine decarboxylase as an early, possibly obligatory, event in mouse skin carcinogenesis.. Cancer Res.

[OCR_00372] Olson J. W., Russell D. H. (1979). Prolonged induction of hepatic ornithine decarboxylase and its relation to cyclic adenosine 3':5'-monophosphate-dependent protein kinase activation after a single administration of diethylnitrosamine.. Cancer Res.

[OCR_00388] Pamukcu A. M., Yalçiner S., Hatcher J. F., Bryan G. T. (1980). Quercetin, a rat intestinal and bladder carcinogen present in bracken fern (Pteridium aquilinum).. Cancer Res.

[OCR_00394] Price J. M., Pamukcu A. M. (1968). The induction of neoplasms of the urinary bladder of the cow and the small intestine of the rat by feeding bracken fern (Pteris aquilina).. Cancer Res.

[OCR_00400] Russell D., Snyder S. H. (1968). Amine synthesis in rapidly growing tissues: ornithine decarboxylase activity in regenerating rat liver, chick embryo, and various tumors.. Proc Natl Acad Sci U S A.

[OCR_00407] Scalabrino G., Pösö H., Hölttä E., Hannonen P., Kallio A., Jänne J. (1978). Synthesis and accumulation of polyamines in rat liver during chemical carcinogenesis.. Int J Cancer.

[OCR_00413] Takano S., Matsushima M., Ertürk E., Bryan G. T. (1981). Early induction of rat colonic epithelial ornithine and S-adenosyl-L-methionine decarboxylase activities by N-methyl-N'-nitro-N-nitrosoguanidine or bile salts.. Cancer Res.

